# Human seasonal influenza under COVID-19 and the potential consequences of influenza lineage elimination

**DOI:** 10.1038/s41467-022-29402-5

**Published:** 2022-03-31

**Authors:** Vijaykrishna Dhanasekaran, Sheena Sullivan, Kimberly M. Edwards, Ruopeng Xie, Arseniy Khvorov, Sophie A. Valkenburg, Benjamin J. Cowling, Ian G. Barr

**Affiliations:** 1 School of Public Health, LKS Faculty of Medicine, The University of Hong Kong, Hong Kong, China; 2 HKU-Pasteur Research Pole, School of Public Health, LKS Faculty of Medicine, The University of Hong Kong, Hong Kong, China; 3 WHO Collaborating Centre for Reference and Research on Influenza, VIDRL, Peter Doherty Institute for Infection and Immunity, 3000 Melbourne, VIC Australia; 4 Department of Microbiology and Immunology, University of Melbourne, 3000 Melbourne, VIC Australia

**Keywords:** Molecular evolution, Epidemiology, Influenza virus, Policy and public health in microbiology, Influenza virus

## Abstract

Annual epidemics of seasonal influenza cause hundreds of thousands of deaths, high levels of morbidity, and substantial economic loss. Yet, global influenza circulation has been heavily suppressed by public health measures and travel restrictions since the onset of the COVID-19 pandemic. Notably, the influenza B/Yamagata lineage has not been conclusively detected since April 2020, and A(H3N2), A(H1N1), and B/Victoria viruses have since circulated with considerably less genetic diversity. Travel restrictions have largely confined regional outbreaks of A(H3N2) to South and Southeast Asia, B/Victoria to China, and A(H1N1) to West Africa. Seasonal influenza transmission lineages continue to perish globally, except in these select hotspots, which will likely seed future epidemics. Waning population immunity and sporadic case detection will further challenge influenza vaccine strain selection and epidemic control. We offer a perspective on the potential short- and long-term evolutionary dynamics of seasonal influenza and discuss potential consequences and mitigation strategies as global travel gradually returns to pre-pandemic levels.

## Abstract (web summary)

COVID-19 control measures have suppressed circulation of other infections including influenza. Here, the authors analyse WHO global influenza sequence and case report data and describe changes in the phylogenetic and geographic distribution of influenza lineages during the COVID-19 pandemic.

## Introduction

Seasonal influenza viruses evolve to evade pre-existing immunity and gain competitive advantage via surface protein mutations which yield new antigenic variants^1^. Natural selection acts on a global scale due to rapid and widespread global circulation^2^. This effectively eliminates previously dominant antigenic variants and results in limited circulation of antigenically similar viruses within each subtype/lineage at a given point in time. However, the pace of antigenic selection varies over time for influenza A virus (IAV) subtypes and influenza B virus (IBV) lineages due mainly to population-level fluctuations in immune pressure. This confounds vaccine strain selection, which relies on the prediction of antigenic evolution^3^. To facilitate bi-annual selection of candidate vaccine viruses, the WHO Global Influenza Surveillance and Response System (GISRS) coordinates influenza surveillance from 138 National Influenza Centers (NICs) and diagnostic and reference laboratories in 108 countries^4^. Current seasonal vaccine formulations are either trivalent or quadrivalent, with either three or four representative strains including IAV subtypes A(H1N1) and A(H3N2) and either one or both IBV lineages, B/Victoria and B/Yamagata.

Seasonal influenza viruses exhibit stronger seasonal cycles in temperate zones, with surges of infections in winter. Seasonal trends are weaker in tropical zones, with increased circulation evident in both the rainy season due to increased humidity and in cooler, drier months^5,6^. Seasonal temperate cycles are maintained through continuous reintroduction from tropical regions and opposing hemispheres, causing local transmission chains to emerge and perish in community settings^7,8^. Transmission chains arising from a single introduction (transmission lineages^9^) dissipate at a greater frequency outside of peak seasonal circulation, although some may persist from one season to the next^2,10^. In tropical regions, influenza viruses exhibit more complex multi-peak dynamics, impacted by patterns of global circulation and evolution^11^. The interplay between the different seasonal influenza virus subtypes and lineages varies temporally and geographically, leading to significant variation in population immunity to each influenza virus.

**Fig. 1:**
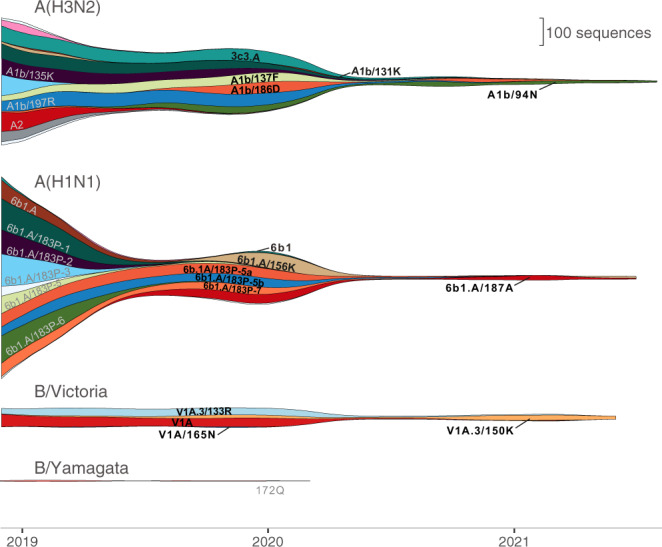
Streamgraph showing temporal changes in influenza lineage circulation. Lineage prevalence was estimated using sample collection dates of all sequences submitted to the Global Initiative for Sharing All Influenza Data (GISAID) from December 2018 to August 2021. Lineages detected since April 2020 are labeled in black; lineages that have not been detected since April 2020 are labeled in gray.

Analysis of global sequence data has shown that (i) tropical and subtropical regions in Asia sustain transmission lineages for a longer duration than temperate regions, providing more opportunities for antigenic drift^2^, and (ii) A(H3N2) lineages do not generally persist between seasons in temperate regions but are reseeded annually^7,8,12^, whereas transmission lineages of A(H1N1), B/Yamagata, and B/Victoria can circulate for several years in temperate and sub-tropical regions^2^. Population density and regional interconnectedness play an important role in maintaining viral metapopulations^8,12^. However, the genetic and antigenic diversity of seasonal influenza has been severely impacted by dramatic changes in global migration and travel since the onset of the COVID-19 pandemic in March 2020 (Fig. 1).

Since April 2020, most countries have seen historically low seasonal influenza virus circulation^13,14^ attributable to non-pharmaceutical interventions (NPIs) such as travel restrictions, quarantine on arrival, social distancing, school and workplace closures, mask wearing, surface disinfection, and enhanced hand hygiene. NPIs have similarly disrupted the circulation of other common respiratory viruses such as respiratory syncytial virus and human metapneumovirus^15–18^ by limiting opportunities for reintroduction and local transmission. Prolonged suppression of seasonal influenza virus circulation, compounded by regional inequities in vaccine distribution^19^ and potential vaccine complacency, supply chain disruptions and misinformation^20^ amid fewer cases, will reduce population immunity and increase severity of future influenza virus epidemics. Accumulating evidence indicates protection against influenza infection, acquired through infection or vaccination, wanes over the course of a single season^21^. At the individual level, circulating antibodies decline over six months^22^, and the half-life of T-cells for cellular responses lasts eight to 14 years^23^. Accumulation of susceptible individuals during milder seasons results in more intense subsequent seasonal epidemics^24^. The consequences may be most dire for children with lack of exposure to influenza, as immunological imprinting (also referred to as “original antigenic sin”) during childhood influenza A and B infections^25–27^ impacts patterns of susceptibility and circulation in subsequent years^18^. Epidemiological studies, corroborated by multiple modeling and immunological studies^27,28^, show lifelong immune memory to first childhood influenza infection confers lifelong homosubtypic protection at the cost of heterosubtypic protection. Prolonged suppression of seasonal influenza circulation during the 2020s will lead to greater susceptibility in this birth cohort due to lack of exposure by natural infection.

As COVID-19 vaccination rates increase in the coming months, the use of NPIs to limit transmission will gradually decline. Domestic and international travel will eventually return to pre-pandemic levels^29^, enabling a resurgence of influenza virus circulation. Through phylogenetic analysis of available influenza sequence data and case reports submitted to WHO GISRS we consider the short- and long-term implications of COVID-19 control measures on the epidemiology and evolution of seasonal influenza viruses.

## Results

### A global reduction in seasonal influenza virus case detection

**Fig. 2:**
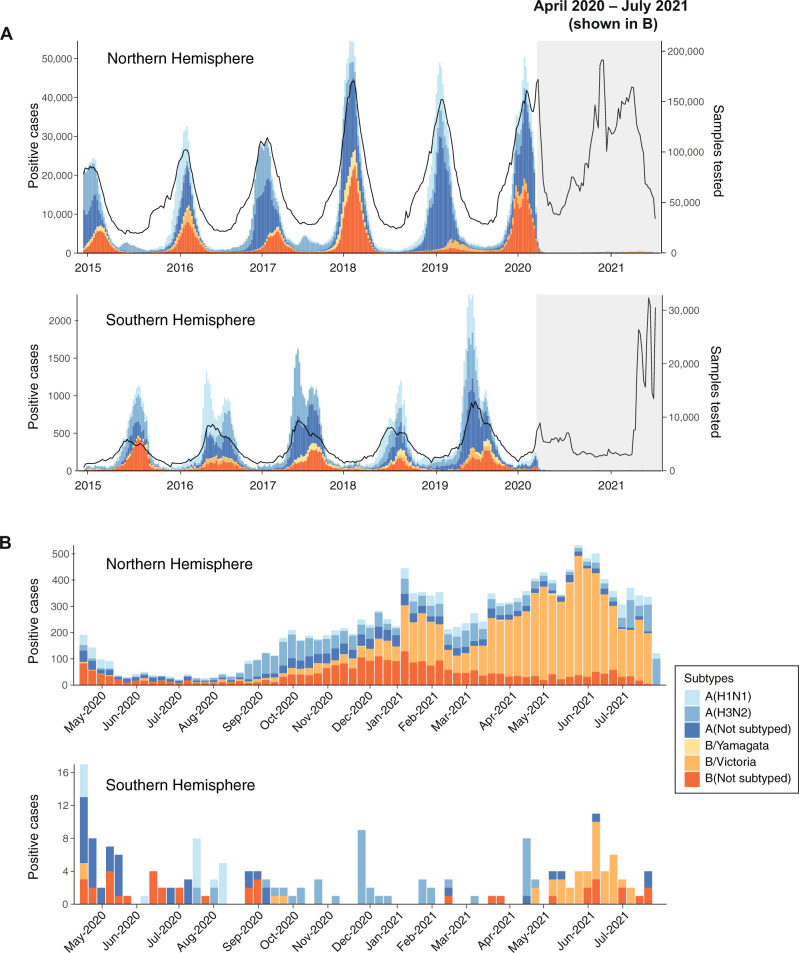
Virological surveillance of seasonal influenza viruses. **A** Time series comparing FluNet data on seasonal influenza activity in the Northern and Southern Hemispheres from 2015 to July 2021, with the COVID-19 pandemic shaded in gray. Stacked bar chart (left-hand *y*-axis) represents the number of influenza-positive cases per week colored by subtype. Black trend-line (right-hand *y*-axis) shows the number of specimens tested per week. **B** Magnified view of the gray-shaded bar charts in **A** showing influenza-positive specimens from April 2020 to July 2021. Note: *y*-axis scales differ in each panel.

Analysis of the GISRS FluNet database^4^ to 1 August 2021 shows an unprecedented global reduction in seasonal influenza cases since the beginning of the COVID-19 pandemic in March 2020 (Fig. 2). Routine influenza testing was disrupted during the initial stages of the pandemic amid the high demand for SARS-CoV-2 testing. Nevertheless, many countries continued or resumed influenza testing and reporting by mid-2020^15,30,31^, and the dramatic decline in influenza virus detection cannot be explained by this transient disruption in laboratory testing. During the 2017/2018 to 2019/2020 Northern Hemisphere winter seasons, the number of influenza positive cases peaked around 40,000–60,000 per week. In early February 2020, cases in the Northern Hemisphere fell from a peak of ~50,000 cases per week to <100 cases per week in May 2020 and remained below 100 weekly cases until September 2020, a 99.8% reduction (Fig. 2). During the first half of 2021, case numbers increased marginally to 200–400 cases per week. Similarly, in the Southern Hemisphere, activity during the 2017–2019 seasons peaked between 1500 and 3500 positive specimens per week, but the 2020 season was notably absent and the expected rise in seasonal influenza cases has yet to occur in 2021. Remarkably, <12 influenza positive cases per week were reported from May 2020 to July 2021 in the Southern Hemisphere (Fig. 2).

### Reduction in seasonal influenza virus diversity and the likely elimination of B/Yamagata

**Fig. 3:**
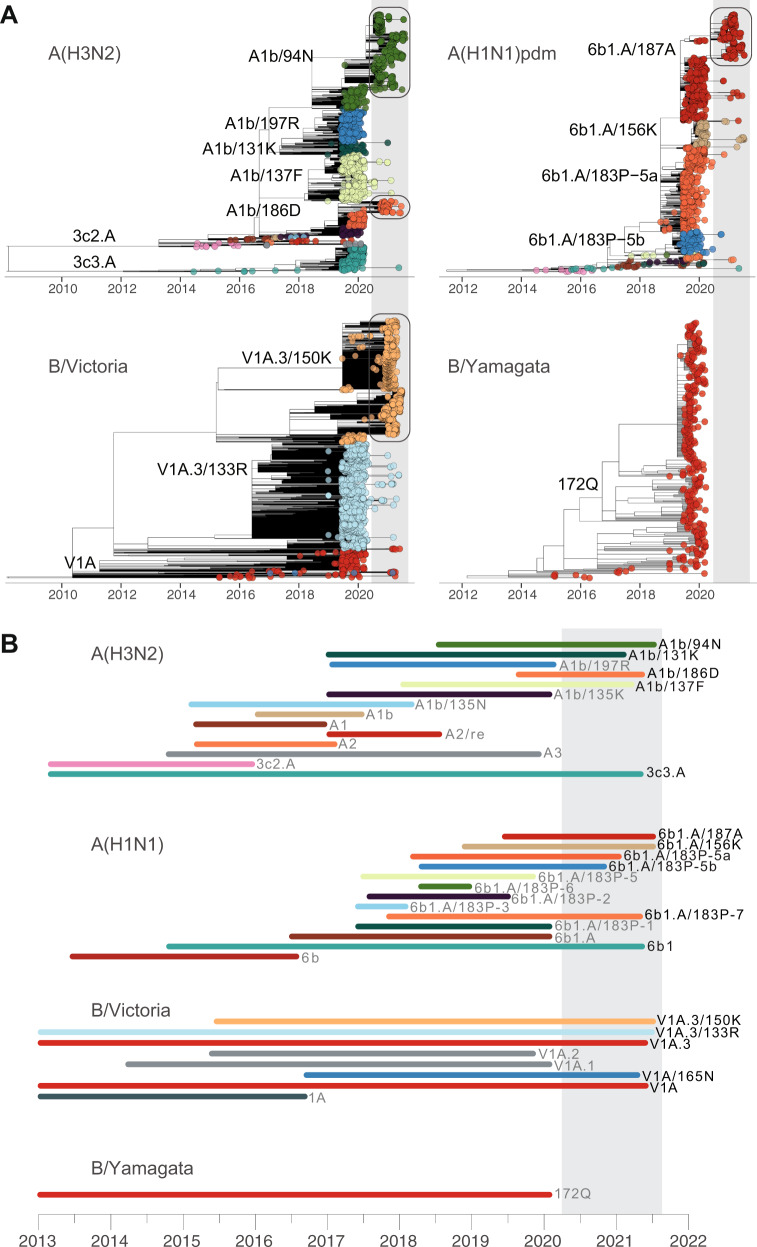
Comparison of seasonal influenza virus evolution before and since COVID-19 emergence. **A** Evolutionary relationships and divergence times of *HA* genes inferred using maximum likelihood (see Online Methods section). Tips are colored by WHO clade designations. Phylogenetic trees of major clades that continued to circulate during 2020/2021 (marked in **A**) are shown in Supplementary Figs. 1–4, including A(H3N2) clades A1b/94N and A1b/186D; A(H1N1) clade 6b1.A/187A lineages in West Africa; and B/Victoria lineages from several regions around the world. **B** Timeline of recently circulating seasonal influenza virus *HA* clades from mean estimated divergence time to most recent sequence. Time since the onset of the COVID-19 pandemic is shaded in gray.

Co-circulation of diverse A(H3N2) and B/Victoria viruses and antigenic drift within some co-circulating clades have necessitated frequent updates to the vaccine strain components in recent years. Since their emergence in 1968, A(H3N2) viruses have, on average, evolved distinct antigenic variants every 3–7 years with rapid elimination of previous antigenic variants^32,33^. However, leading up to the COVID-19 pandemic, a major A(H3N2) genetic bottleneck had not occurred for a number of years^34^ (Figs. 1 and 3). The continued circulation of an A(H3N2) clade 3c3.A, a lineage which dates back to 2013, has been implicated in reduced production of neutralizing antibodies in adults with childhood exposure to A(H3N2) ^28^, and it has been hypothesized that further accumulation of antigenic changes may result in A(H3N2) divergence^35^. Like A(H3N2), the genetic diversity of B/Victoria expanded from 2015 to 2018, with seven subclades co-circulating prior to the COVID-19 pandemic, albeit with less antigenic diversity than A(H3N2). In contrast, the 2009 pandemic A(H1N1) viruses have shown slower antigenic drift, with 6b1/183P-5a as the dominant A(H1N1) clade amid a number of antigenically related subclades, and B/Yamagata viruses have exhibited weak antigenic selection in recent years, further reducing their prevalence over time^36^.

From April 2020 through July 2021, only 2521 influenza A(H3N2) virus cases were reported from 57 countries in FluNet (Supplementary Dataset 1), with outbreaks apparent in West Africa (Côte d’Ivoire (*n* = 123) and Senegal (*n* = 119)), South Asia (Bangladesh (*n* = 209), India (*n* = 455), Pakistan (*n* = 162), and Nepal (*n* = 107)), and Southeast Asia (Cambodia (*n* = 108), Laos (*n* = 268), and Vietnam (*n* = 162)). Only 590 A(H3N2) sequences from this 16-month period were deposited in GISAID, *a* > 97% reduction in A(H3N2) sequence data globally in comparison to the previous 16 months. Of the eight A(H3N2) subclades that circulated during 2019/2020, three have not been detected since April 2020 (A3, A1b/135K, and A1b/197R), while 3c3.A, A1b/94N, A1b/186D, A1b/131K, and A1b/137F continued to circulate in 2021 (Figs. 1 and 3).

**Fig. 4:**
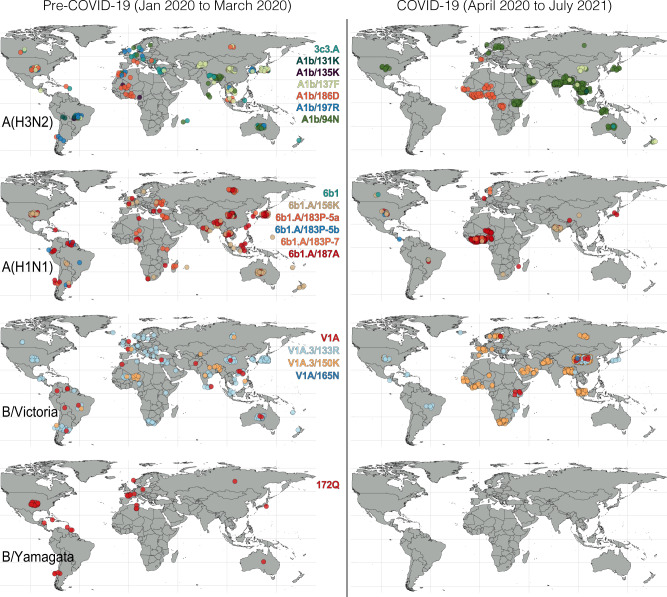
Geographic distribution of influenza *HA* sequences before (left) and after (right) COVID-19 emergence. From April 2020 to July 2021, A(H3N2) (590 sequences from 32 countries), A(H1N1) (254 sequences from 18 countries), and B/Victoria (834 sequences from 34 countries) were available for analysis. In comparison to the 16-month period before April 2020 (December 2018 to March 2020), there was a reduction in sequences of 97% for A(H3N2), 99% for A(H1N1), 92% for B/Victoria, and 100% for B/Yamagata. Last updated on 24 August 2021; for an interactive visualization of current seasonal influenza circulation, we refer the reader to the Nextstrain^67^ platform.

Maximum likelihood phylogenetic analysis of seasonal influenza virus hemagglutinin (*HA*) sequences since SARS-CoV-2 emergence illustrates the circulation of geographically and genetically distinct A(H3N2) outbreaks in parts of West Africa, South Asia, and Southeast Asia (Fig. 4 and Supplementary Dataset 2). Clade A1b/137F viruses were detected sporadically in Bangladesh, Laos, New Zealand, United Arab Emirates, and Yunnan province in China (Fig. 4 and Supplementary Dataset 2). A(H3N2) clade A1b/186D was detected primarily in West Africa (Supplementary Fig. 1), while clade A1b/94N was detected across Asia and Oceania (Supplementary Fig. 2). In particular, A1b/94N viruses were frequently detected in India since May 2021. The phylogeny of clade A1b/94N reveals six related clusters that originated independently prior to March 2020. First detected in Cambodia, one lineage circulated in the Greater Mekong sub-region of Southeast Asia from July to February 2020. A second distinct A1b/94N lineage was detected in the Australian Northern Territory from individuals returning to Australia and in quarantine during February–March 2021 and from neighboring Timor-Leste during July 2020–March 2021, suggesting regional circulation during 2020/2021 (Supplementary Fig. 2). Three other A1b/94N lineages, represented by only one or two identical *HA* sequences, were detected in Japan on 2 March 2021, Bangladesh on 28 September 2020, and Côte d’Ivoire on 24 November 2020. While most A1b/94N lineages have been regionally contained, one A1b/94N lineage with common ancestry dating back to 2019 in South Asia has been detected in India, Bangladesh, United Arab Emirates, Australia, Kenya, Singapore, the United States, and several European countries during 2020 and early 2021 (Figs. 3A and 4, Supplementary Fig. 2 and Supplementary Dataset 2).

Few A(H1N1) cases have been detected since April 2020 (Fig. 2), mainly in Ghana (*n* = 235), Togo (*n* = 226), the United States (*n* = 170), and Russia (*n* = 165) (Supplementary Dataset 1). Nevertheless, the 254 available sequences in GISAID reflect cryptic circulation of all previously circulating A(H1N1) clades into early 2021. Three independent lineages of clade 6b1.A/187A viruses circulated in West Africa (Ghana, Nigeria, and Togo) during 2020, along with a few 6b1.A/156K and 6b1.A/183P-5a viruses (Fig. 4 and Supplementary Fig. 3). Since May 2021, clade 6b1.A/156K viruses were primarily detected in India, while the other A(H1N1) clades in circulation have been detected sporadically around the world (Fig. 4).

An ongoing B/Victoria epidemic in China accounts for the majority of all seasonal influenza viruses detected in 2020/2021 globally (Supplementary Dataset 1). Since November 2020, China has consistently detected over 400 B/Victoria cases per week. While B/Victoria clades V1A.1 and V1A.2 have not been reported for over 16 months, five other B/Victoria clades (V1A, V1A.3, V1A.3/133R, V1A.3/150K, and V1A/165N) continue to circulate (Fig. 3). Phylogenetic analysis shows the circulation of two distinct lineages of clade V1A.3/150K since late 2020 (Supplementary Fig. 4). Initially detected in China in September 2019, one V1A.3/150K lineage has been intermittently detected in other parts of the world up to March 2020. The other circulating lineage of clade V1A.3/150K has been detected since late 2020 in parts of China, Bangladesh, West Africa, the Middle East, Europe, Russia, and most recently in Singapore during June 2021 (Fig. 4 and Supplementary Dataset 2). Several highly similar *HA* sequences were detected in West Africa, the Middle East, and Europe, suggesting limited intercontinental transmission of V1A.3/150K viruses has resumed (Supplementary Fig. 4). The B/Victoria clade that was dominant prior to March 2020 globally, V1A.3/133R, was infrequently detected from April 2020 to May 2021, mainly from China, Kenya, the United States, Brazil, and Japan (Fig. 4 and Supplementary Dataset 2). Similarly, clade V1A B/Victoria-lineage viruses, which circulated globally in 2019, were only detected small numbers in China, Kenya, and Sweden since April 2020 (Supplementary Dataset 2). Clade V1A/165N has been reported infrequently since 2017, and the most recent sequence was detected in Zhejiang province in China on March 2021, suggesting low-level circulation of this B/Victoria clade. In summary, V1A.3/150K has been detected globally, especially in China, Bangladesh, and Singapore during recent months and will likely continue to dominate. Other B/Victoria clades (e.g., V1A, V1A.3/133R, V1A/165N) have been regionally detected, albeit at lower frequencies, since April 2020 and may eventually be replaced by descendants of the apparently fitter V1A.3/150K viruses.

Notably, in February 2022, WHO reported that no confirmed B/Yamagata detections had been reported since March 2020^37^, which suggests transmission of B/Yamagata has not been sustained.

### Transmission dynamics and geographic hotspots of seasonal influenza virus circulation

**Fig. 5:**
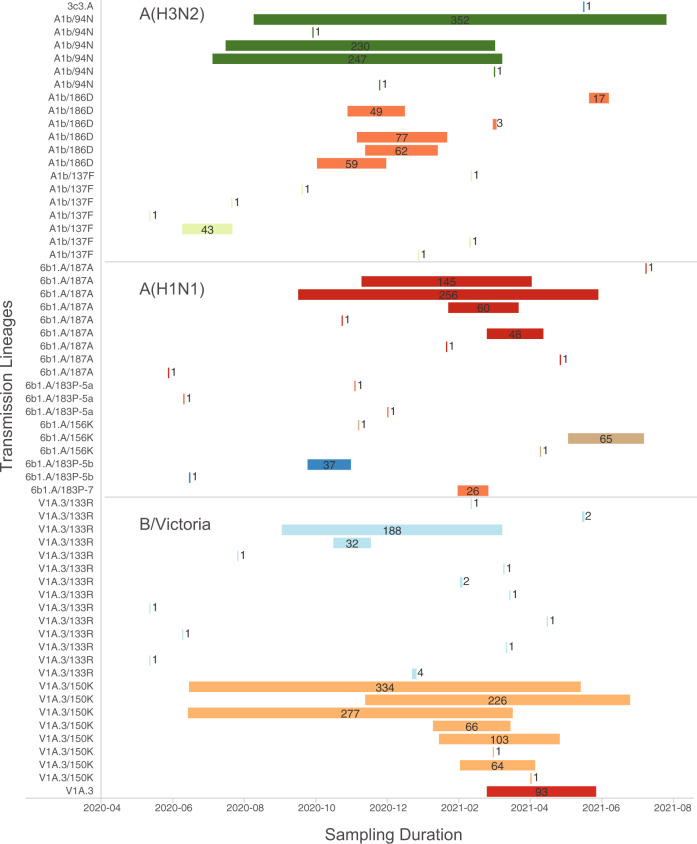
Cluster size and duration of influenza transmission lineages that originated before the COVID-19 pandemic. Each colored bar represents a monophyletic transmission lineage inferred from maximum-likelihood phylogenetic analyses of seasonal influenza *HA* gene sequences in GISAID (see Online Methods section). Labels indicate sequence counts per transmission lineage.

We generated large-scale phylogenetic trees of *HA* sequence data from 2018–2021 to determine the number of independent lineages that originated from viruses circulating during March 2020. Over 60 residual transmission lineages were detected in both IAV and B/Victoria (52% singleton sequences), a majority of which occurred amongst the major clades described above (A(H3N2) A1b/94, A(H1N1) 6b1.A/187A, and B/Victoria V1A.3/150K) (Fig. 5). Most residual transmission lineages were derived from viruses circulating within the same country, province, or geographic region (Fig. 4, Supplementary Figs. 1–4 and Supplementary Dataset 3). These results affirm the lack of global dissemination of seasonal influenza viruses during the COVID-19 pandemic and reveal smaller regions with high population densities that can independently sustain influenza virus transmission lineages for extended periods. Furthermore, while detection of A(H3N2) and B/Victoria viruses derived from pre-COVID-19 influenza virus lineages increased during the first two months of 2021, new detections have since decreased, suggesting many residual transmission chains may have terminated at the end of the 2020/2021 Northern Hemisphere season. Two major lineages continue to be detected in mid-2021, B/Victoria V1A.3/150K was most prevalent, and to a lesser extent, A(H3N2) A1b/94N (Supplementary Dataset 2 and Supplementary Figs. 2 and 4).

A(H1N1), A(H3N2), and B/Victoria viruses detected during late 2020–early 2021 largely circulated in West and Central Africa. These outbreak reports are supported by genetic sequences, and many of the observed transmission lineages have been regionally maintained since before COVID-19. According to the COVID-19 control policies of individual countries^38^, internal movement restrictions were enacted across all countries in West Africa by 13 April 2020, and international travel restrictions were in place across the region by 30 March 2020 (Supplementary Fig. 5). However, by late July 2020, most countries in West Africa had lifted domestic travel restrictions, and by August 2020 international travel was allowed with some restrictions. A(H3N2) *HA* gene phylogenies suggest these control measures effectively restricted cross-border spread of influenza viruses, because sequences from Cameroon, Niger, Nigeria, and the Democratic Republic of Congo to January 2021 cluster by country, suggesting containment of virus circulation within each country (Supplementary Fig. 1). In contrast, most Southeast Asian nations maintained relatively stringent domestic and international travel restrictions during 2020/2021, except for Laos and Cambodia, where COVID-19 suppression was followed by A(H3N2) influenza virus outbreaks in 2020. While international travel measures impacted influenza virus migration patterns, an analysis of control-measure stringency and influenza case reports in fourteen countries in Asia and Africa with substantial influenza activity showed no correlation between the stringency of public health interventions and domestic transmission of influenza (see Methods section, Supplementary Fig. 6). A caveat to note is that the stringency index does not reflect the efficacy of control measures or population compliance.

## Discussion

Since the start of the COVID-19 pandemic, WHO influenza surveillance data reflect a substantial reduction in global influenza virus circulation. Lack of exposure to influenza will lower population immunity and increase the severity of large epidemics upon a future global resurgence. Notably, countries in North America and Europe with strong influenza surveillance have only sporadically reported the influenza viruses in circulation, including several that have caused outbreaks in Africa and Asia, and B/Yamagata lineage viruses appear to have become extinct around mid-2020. Despite an overall increase in influenza surveillance, WHO reports^39^ variable effects due to COVID-19. Influenza surveillance benefited due to rapid capacity building and training efforts to respond to SARS-CoV-2, however disruptions occurred at national or regional levels due to healthcare resource allocation and health care-seeking behavior. Generally, demographic details such as age are not available for all countries, many cases are missed due to timing of infection, and severe cases requiring primary care or hospitalization are more likely to be detected and reported. Furthermore, reference laboratories use various detection methods and may only submit a representative subset of their surveillance data alongside any cases that cannot be subtyped by conventional methods. Not all cases are confirmed by isolation or genomic characterization, and of those sequenced, only a portion are submitted to sequence databases such as GISAID. However, to offset issues with completeness of submitted record^40^, our analysis was limited to the primary data fields, and regional circulation was inferred using a combination of case numbers and virus genetic relationships.

Roughly one-quarter or more of seasonal influenza cases are caused by IBVs^41^, and in recent decades the two IBV lineages have caused comparable proportions of influenza cases. Historically, B/Yamagata viruses have caused a greater rate of infection in temperate regions and have infected adults at a greater rate than children, whereas B/Victoria viruses have infected more children than adults^41^. However, the long-term impact of B/Yamagata elimination on the evolutionary dynamics of IBV is uncertain. Recently, Vieira et al.^24^ examined historical patterns of IBV lineage frequencies in New Zealand using statistical modeling and showed that fluctuations in lineage dominance and lineage cross-protection explains contrasting age distributions of B/Yamagata versus B/Victoria lineages. As IBV lineages offer some cross-protection^42,43^, the extinction of B/Yamagata will leave a higher proportion of individuals susceptible to IBV, enabling faster B/Victoria antigenic evolution.

**Fig. 6:**
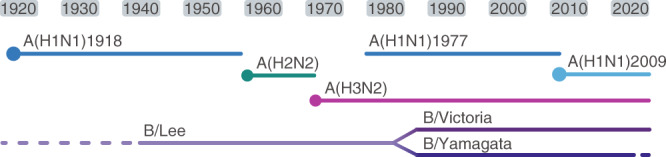
Historical circulation of influenza viruses in the last century. Dots indicate emergence of pandemic strains that replaced previously circulating influenza viruses.

It is important to note, the threat of re-introduction of apparently extinct influenza virus lineages could still pose a risk in coming years, as happened with the reemergence of A(H1N1) in 1977^44^ following a 19-year hiatus since the 1958 A(H2N2) pandemic (Fig. 6). If B/Yamagata does not reemerge in the next year or so, it may need to be treated as a high consequence pathogen to prevent reintroduction, similar to A(H2N2) viruses which have not circulated since 1968 and are now held and handled in the higher level BSL-3 laboratory biosecurity levels^45^. Future B/Yamagata positive samples will require urgent confirmation and characterization to be able to better determine the mechanisms that could sustain such low levels of virus circulation – for example, immunocompromised individuals can carry infection for several weeks or months and potentially accumulate additional mutations^46–48^ – or to rule out the possibility that these were in fact false-positive test results.

Although two IAV subtypes and two IBV lineages have co-circulated in recent decades, prior to the re-emergence of A(H1N1) in 1977, only a single IAV subtype and a single IBV lineage circulated among humans (Fig. 6). In the early 1980s, IBV diverged from the ancestral B/Lee lineage into two antigenically distinct lineages^49^. The survival of two IBV lineages is attributed to the geographic isolation of B/Victoria in China in the 1990s, followed by a global resurgence during 2000-2002^50^. The continued endemicity of geographically disparate transmission lineages of A(H3N2), A(H1N1), and B/Victoria (compounded by limited availability of clinical isolates) confounds the accuracy of candidate vaccine virus selection, and further accumulation of antigenic changes could lead to long-term co-circulation of antigenically distinct lineages, as occurred for IBV. However, the concomitant reduction in population-level immunity towards seasonal influenza suggests global resurgence of any residual viruses could occur in the future and continued vigilance is required.

The emergence of pandemic influenza viruses A(H2N2) in 1958 and A(H3N2) in 1968 from animal reservoirs resulted in the rapid and complete elimination of previously circulating seasonal influenza A subtypes (Fig. 6) in part due to a lack of pre-existing immunity, which enables novel strains to out-compete their predecessors. However, while the recent emergence of the 2009 A(H1N1) pandemic virus caused elimination of A(H1N1)77 viruses (last detected in 2010; GISAID), seasonal A(H3N2) and IBV viruses sustained transmission throughout the pandemic. Mild and short-lived NPIs temporarily suppressed circulation of A(H3N2) and IBVs, and a combination of immune-driven selection and relatively slower antigenic evolution of A(H1N1)77^36^ likely contributed to its elimination. By 2011, seasonal circulation of all four subtypes had resumed, though A(H3N2) evolutionary patterns were significantly altered following co-circulation with 2009 A(H1N1) viruses^35^.

A previous analysis of global sequencing data highlighted the propensity for sub-tropical regions in Asia to sustain transmission lineages and act as source populations in the emergence of influenza antigenic variants^2^. However, limited sequence and surveillance data were available from Africa at that time. Surveillance capacity in West Africa has since increased with direct support from the WHO and US CDC. In the context of pandemic disruptions to influenza circulation, surveillance in West Africa highlights the potential importance of this region for sustained transmission of influenza and suggests that this region may play a key role in the circulation and maintenance of seasonal influenza lineages along with larger population centers located in India, China, and Southeast Asia. Furthermore, recent studies that showed correlation between stringency and national-level influenza transmission were from countries with no influenza activity since pandemic emergence^31,51–53^, with the exception of Cambodia that showed A(H3N2) activity during 2020^54^. Our study in contrast analyzed countries with significant influenza activity to find no correlation between the stringency of community measures and domestic influenza transmission indicating effectiveness of community measures in these countries was low.

We speculate that heterogeneity in COVID-19 vaccination rates and control policies will slow the global resurgence of influenza, delay competition among existing influenza lineages and enable further divergence of spatially separated lineages, but these individual influenza lineages will eventually expand, compete, and once again circulate more widely. Upcoming influenza seasons could therefore be compounded in severity as immunity wanes over time for all age groups^24^. Moreover, the continued evolution of regionally distinct lineages increases the risk that the antigens included in the vaccine will not be representative of the viruses that ultimately circulate, thereby reducing vaccine effectiveness.

Knowledge gained from influenza epidemiology and evolution under COVID-19 epidemic control underscores the importance of heightened vigilance and continued influenza vaccination programs as we emerge from the COVID-19 pandemic, as well as the potential consequences of recent changes in seasonal influenza virus lineage diversity. Based on observed genetic diversity and endemicity of circulating lineages, continued travel restrictions will limit the number of regional introductions, and prolonged pandemic mitigation strategies could further impact future seasonal influenza virus circulation and evolution. Ongoing global COVID-19 vaccination rates indicate that middle-income countries may be sufficiently vaccinated by the start of 2022; thus, continuation of mitigation strategies may become impractical, and global travel could return to pre-COVID-19 levels in the near future. As international travel is important for sustaining seasonal influenza transmission^36,55^, genomic surveillance at border crossings (using the infrastructure developed for COVID-19) could monitor importation from regions that maintain endemic circulation of seasonal influenza. As illustrated by influenza sequence and surveillance data from 2020 and 2021, East, South, and Southeast Asia have had sustained A(H3N2) and B/Victoria transmission lineages, and West Africa has maintained A(H1N1) circulation.

The uncertainty in future seasonal influenza circulation provides further incentive for rapid advancement of universal influenza vaccines that confer broad protection against multiple IAV or IBV lineages^56–58^. Indeed, the mRNA vaccine technology used against COVID-19 could be rapidly produced, modified, and deployed^59,60^ with the potential to alleviate many of the concerns presented in this manuscript. Ultimately, regardless of the influenza vaccine technologies deployed and their coverage, surveillance remains the key to better understanding and controlling influenza infections in the immediate future.

## Methods

Epidemiological trends of seasonal influenza-positive cases and samples tested between January 2015 and July 2021 (Fig. 2 and Supplementary Fig. 6) were inferred from influenza notifications submitted to the WHO Global Influenza Surveillance and Response System (GISRS) ^4^, obtained using FluNet-Scraper (https://github.com/MagnusBook/flunet-scraper). All human seasonal influenza hemagglutinin (*HA*) sequences collected from December 2018 to July 2021 were downloaded from GISAID (Supplementary Dataset 4) and aligned by *HA* subtype/lineage using MAFFT v.7.22^61^. Preliminary maximum-likelihood phylogenies were estimated with FastTree v.2.1^62^. Root-to-tip regression analyses of phylogenetic branch lengths and sampling dates were used to control phylogenetic data quality in TempEst v.1.5.3^63^, and sequences <900 nt were excluded. After adding *HA* reference sequences (recommended vaccine strains from 2010 to 2021), the final dataset included 15,526 A(H3N2), 16,020 A(H1N1), 9,743 B/Victoria, and 1029 B/Yamagata sequences.

Phylogenetic relationships and divergence times of seasonal influenza *HA* genes were estimated using IQ-TREE v.2^64^ and the least-square dating method^65^. Large-scale maximum likelihood analyses using all available HA sequence data were generated by FastTree v.2.1^62^ with the generalized time reversible nucleotide substitution model. Branch support was assessed by Shimodaira-Hasegawa test^66^, and lineages were labeled according to WHO clade designations. Residual influenza virus lineages were estimated by counting individual monophyletic clades that derived from branches prior to March 2020. The R package ‘ggstream’ v.0.1 was used to map temporal changes in sampling of seasonal influenza clades, and ‘rworldmap’ v.1.3 was used to plot world maps.

### Reporting summary

Further information on research design is available in the Nature Research Reporting Summary linked to this article.

## Supplementary information

Supplementary Information

Description of Additional Supplementary Files

Supplementary Data 1

Supplementary Data 2

Supplementary Data 3

Supplementary Data 4

Reporting Summary

## Data Availability

The seasonal influenza gene sequences and associated metadata utilized in this study were downloaded from GISAID (accession numbers and acknowledgements are provided in (Supplementary Dataset 4). Details of confirmed influenza cases are available from the web based tool for influenza virological surveillance FluNet (https://www.who.int/tools/flunet).

## Code availability

Code used for the above analysis is available on 10.5281/zenodo.6321464.
