# Supplementary material for: Human seasonal influenza under COVID-19 and the potential consequences of influenza lineage elimination
Source: Nat Commun. 2022 Mar 31;13:1721. doi: 10.1038/s41467-022-29402-5 (PMC8971476; doi:10.1038/s41467-022-29402-5)
Supplement: Supplementary file 1 — Supplementary Information [file 41467_2022_29402_MOESM1_ESM.pdf]

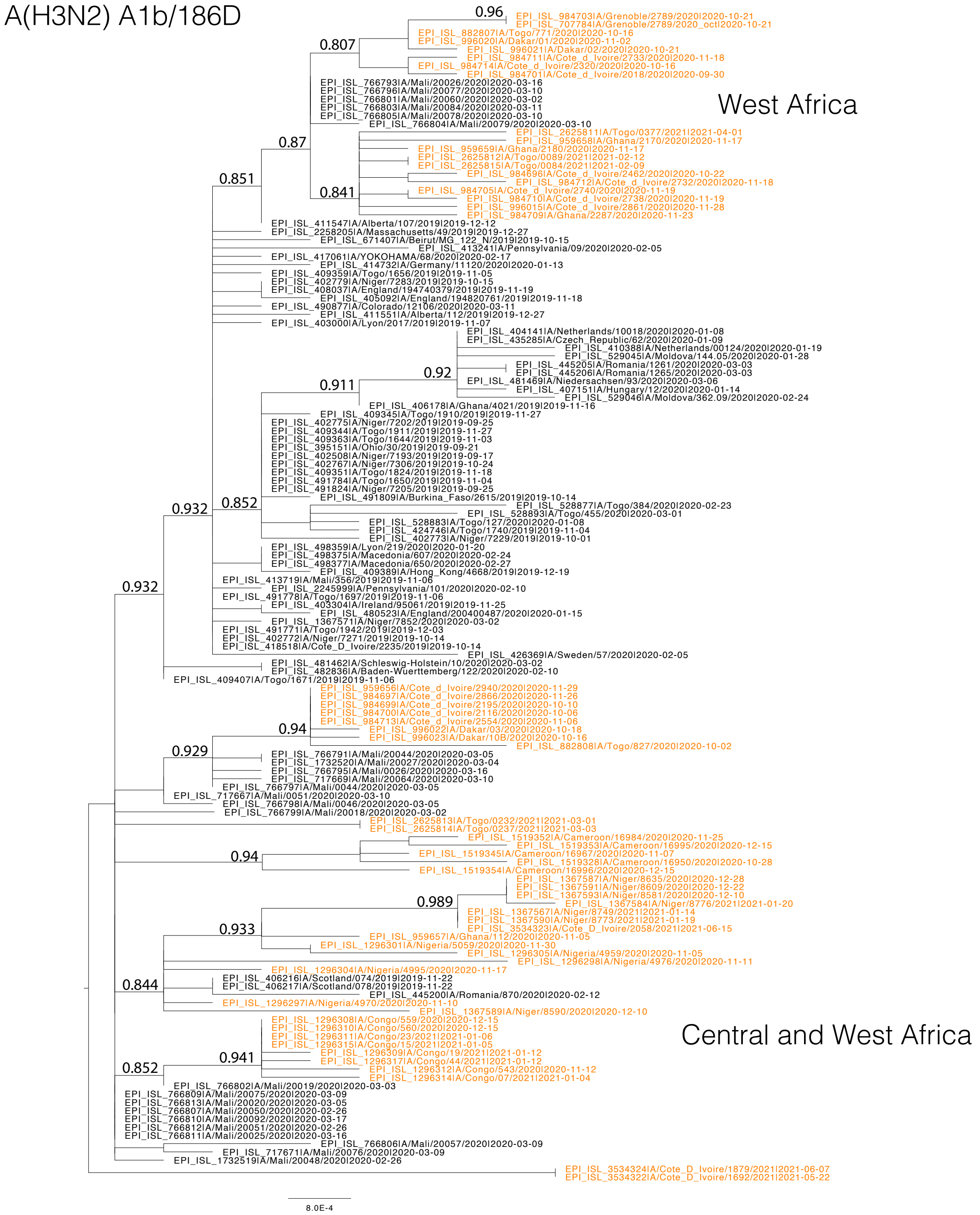

Supplementary Figure 1 | Evolutionary relationships of the HA genes of A(H3N2) A1b/186D clade. Samples collected since April 2020 are colored. Geographic regions with majority of the samples are labelled alongside the tree.The presented tree is a monophyletic lineage that was pruned from a large-scale analysis comprising all available A(H3N2) virus HA sequences collected from January 2018 to August 2021.

A(H3N2) A1b/94N

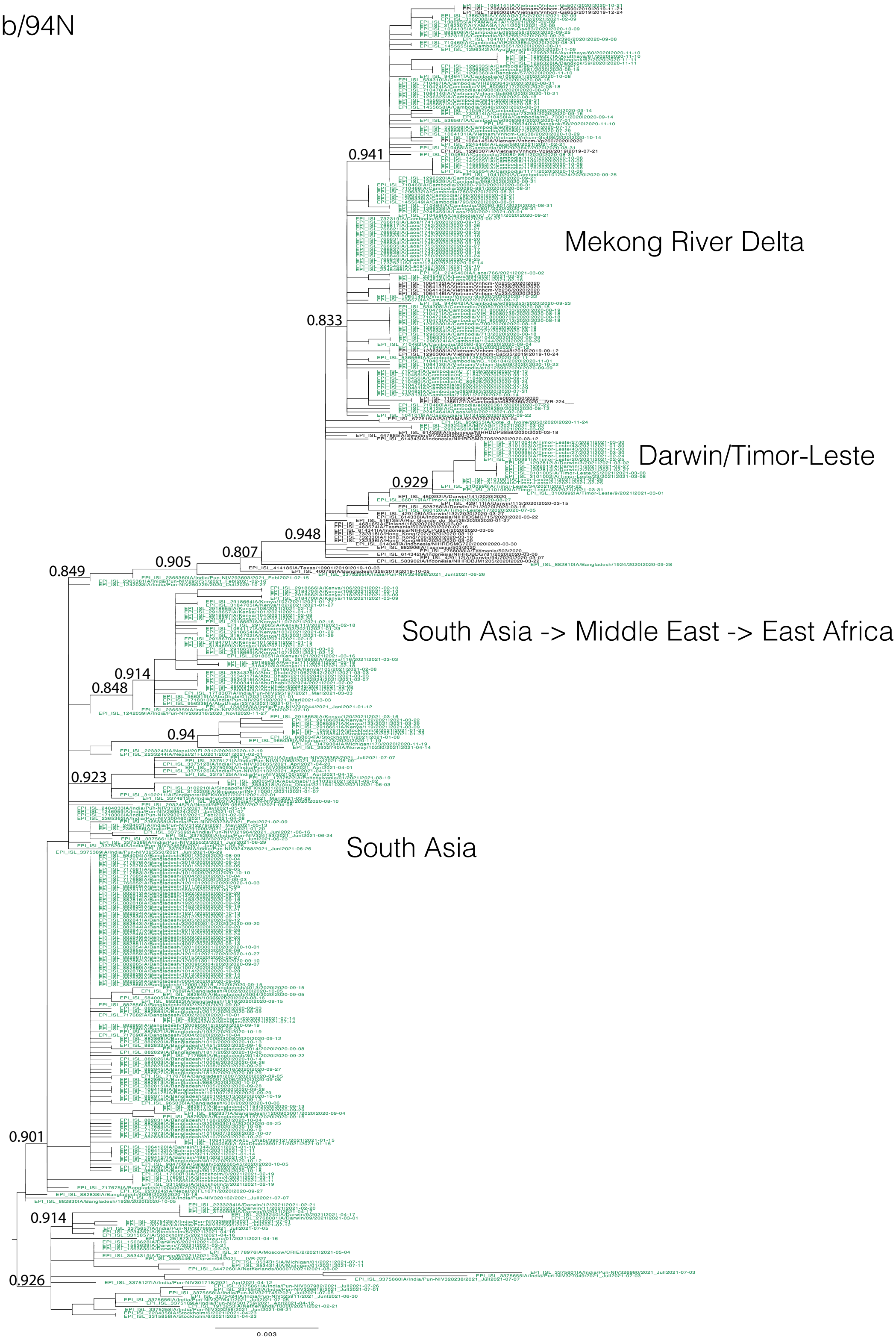

Supplementary Figure 2 | Evolutionary relationships of the HA genes of A(H3N2) A1b/94N clade. Samples collected since April 2020 are colored. Geographic regions with majority of the samples are labelled alongside the tree. The presented tree is a monophyletic lineage that was extracted from a large-scale analysis comprising all available A(H3N2) virus HA sequences collected from January 2018 to August 2021.

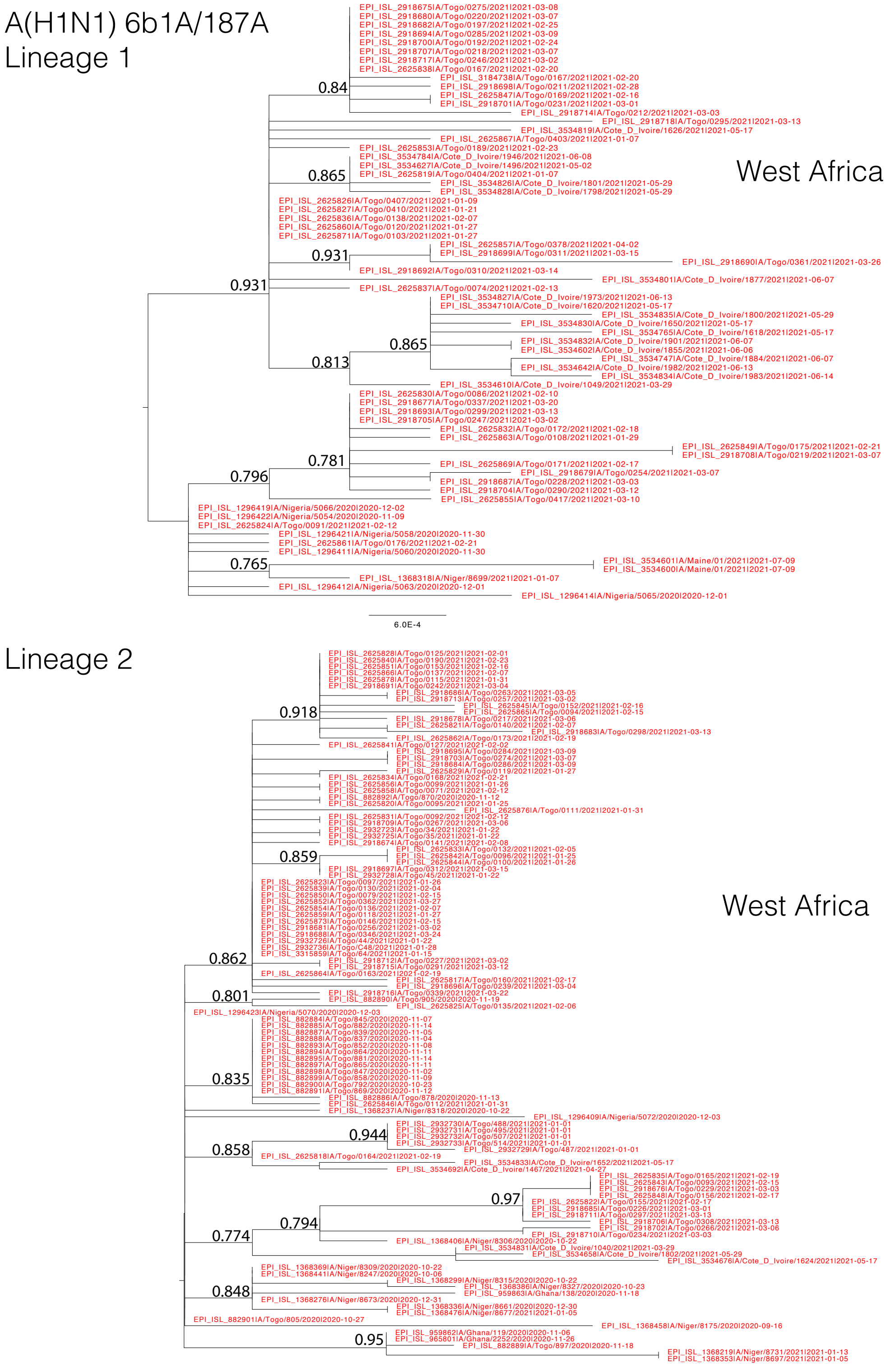

Supplementary Figure 3 | Evolutionary relationships of the HA genes of A(H1N1) 6b1A/187A clade. Samples collected since April 2020 are colored. Geographic regions with majority of the samples are labelled alongside the tree. The presented trees are two closely related monophyletic lineages pruned from a large-scale analysis comprising all available A(H1N1) virus HA sequences collected from January 2018 to August 2021.

Supplementary Figure 4 | Evolutionary relationships of B/Victoria V1A.3/150K clade HA genes. Samples collected since April 2020 are colored. Geographic regions with majority of the samples are labelled alongside the trees. The presented trees are monophyletic lineages pruned from a large-scale phylogeny of all available B/Victoria virus HA sequences collected from January 2018 to August 2021.

B/Victoria V1A.3-150K

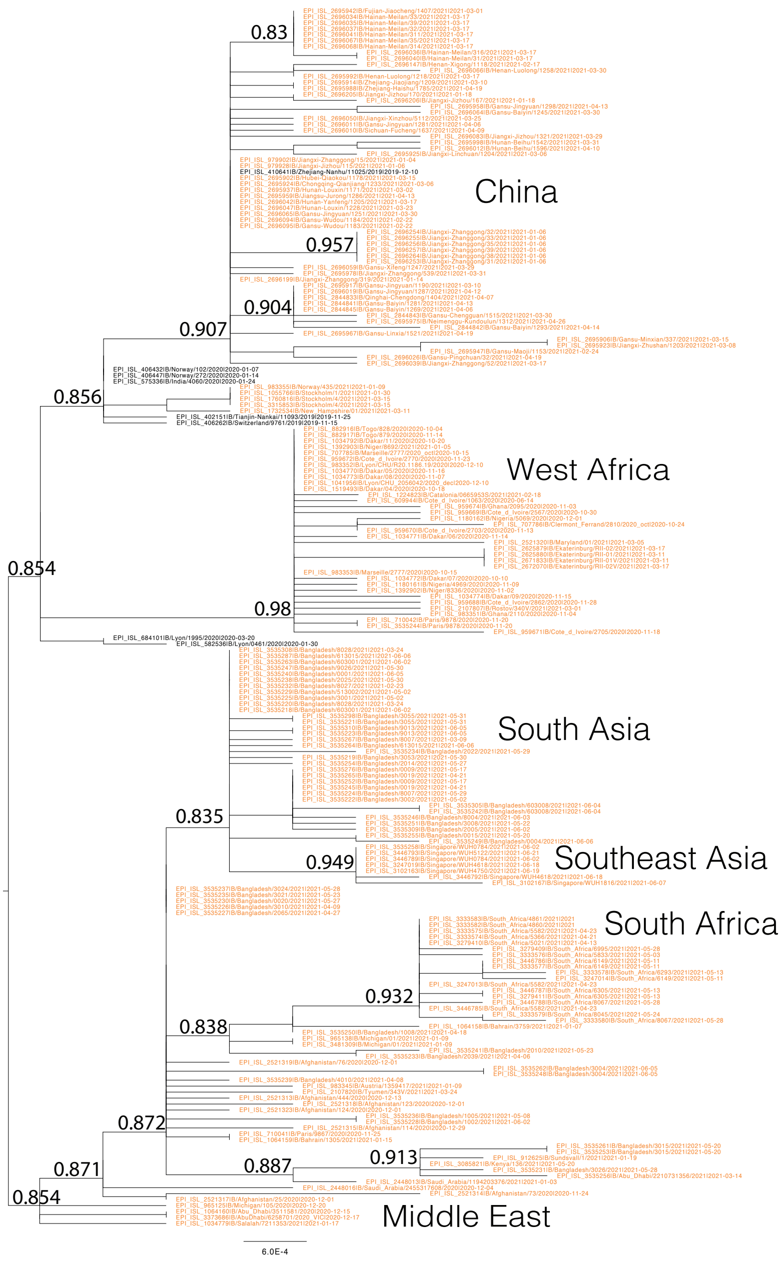

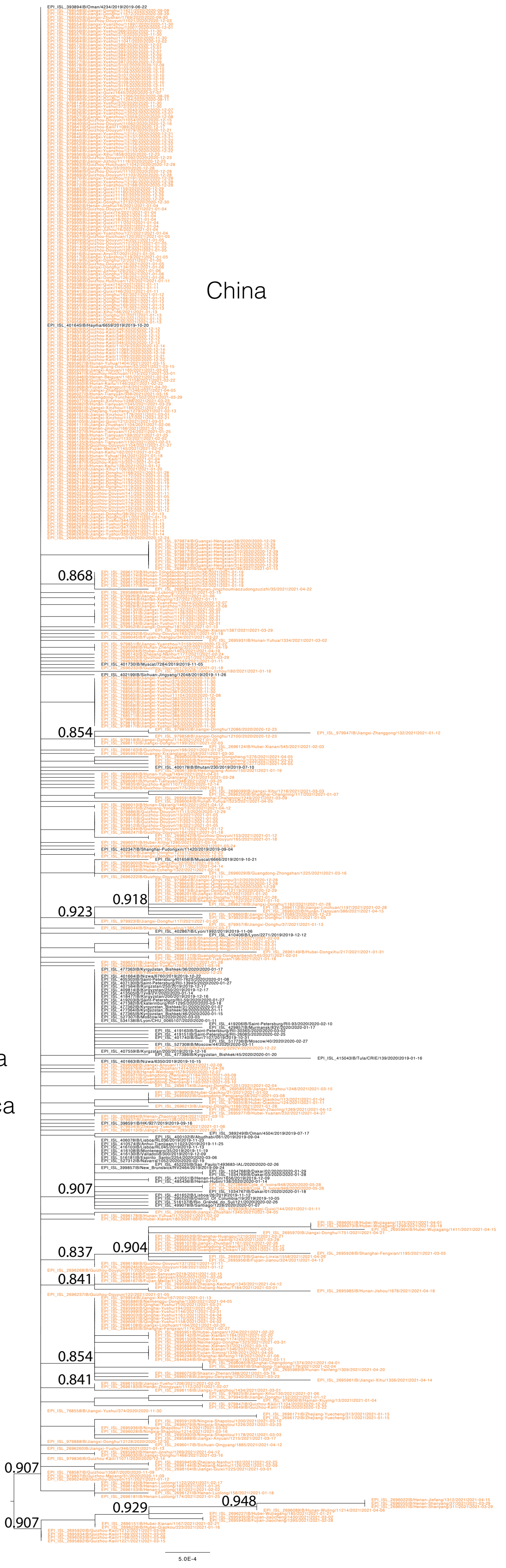

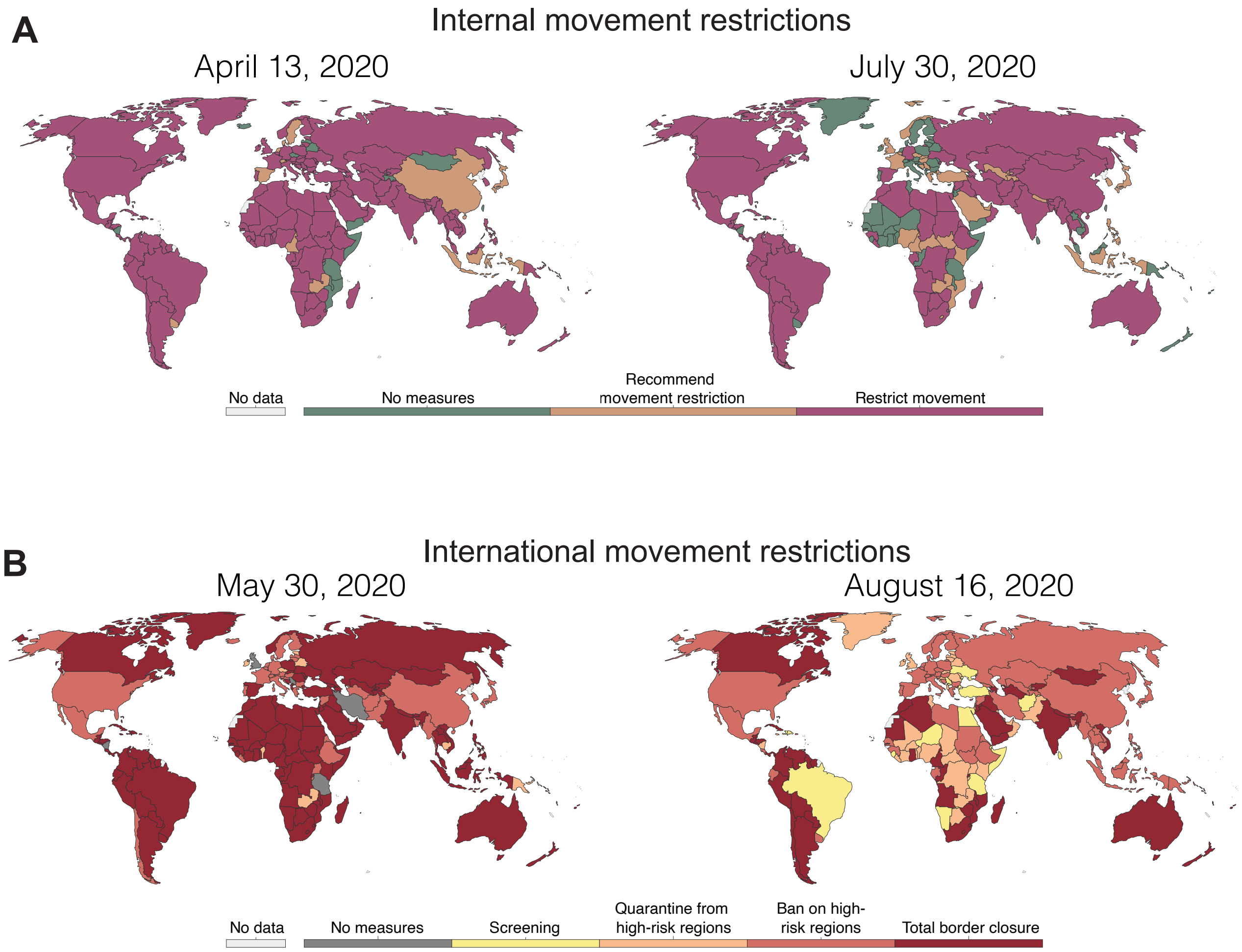

Supplementary Figure 5 | Internal and international movement restrictions enacted by individual countries against COVID-19. Maps were generated by OurWorldinData.org/coronavirus based on the Oxford COVID-19 Government Response Tracker (Hale et al. 2020 *Nature Human Behaviour*)

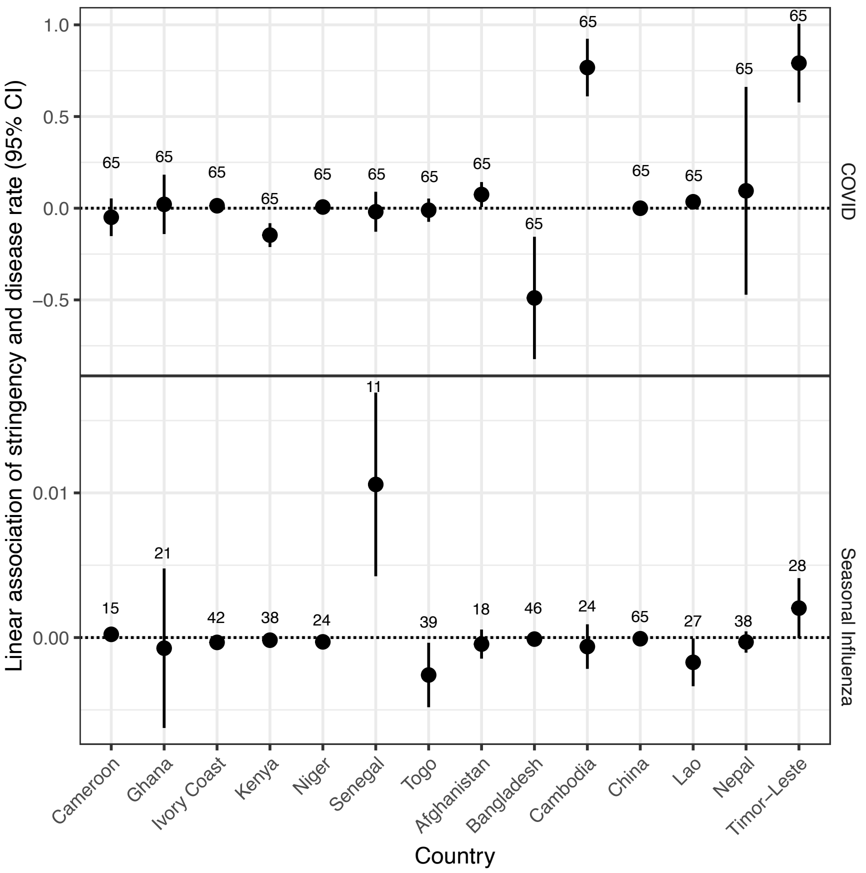

Supplementary Figure 6 | Lack of correlation between the stringency of COVID-19 restrictions and seasonal influenza reports for countries in Africa and Asia with significant seasonal influenza activity. Plots show association between rate (outcome) and stringency (with a two-week lag) as a covariate during 2020-05-01 to 2021-08-01. Points are estimates from the linear model. The number of weeks analysed for each disease-country pair with a rate >0.2 per 100,000 is shown above the points. Intervals are 95% CI for those estimates. Stringency measure is an additive of indicators (school and workplace closures, cancellation of public events, restrictions on gatherings, closings of public transport, public information campaigns, stay at home restrictions, restrictions on internal movement, international travel controls, testing policy, contact tracing, face coverings, vaccination policy) rescaled to vary from 0 to 100. Stringency combines international travel restrictions with other metrics (0 - No measures, 1 – Screening, 2 - Quarantine arrivals from high-risk regions, 3 - Ban on high-risk regions, 4 - Total border closure.) COVID-19 activity was sourced from the WHO COVID-19 Dashboard https://covid19.who.int/ and the multi-sourced Johns Hopkins Coronavirus Resource Center https://coronavirus.jhu.edu/ (labelled JHU).
