# Supplementary material for: Human seasonal influenza under COVID-19 and the potential consequences of influenza lineage elimination
Source: Nat Commun. 2022 Mar 31;13:1721. doi: 10.1038/s41467-022-29402-5 (PMC8971476; doi:10.1038/s41467-022-29402-5)
Supplement: Supplementary file 2 — Description of Additional Supplementary Files [file 41467_2022_29402_MOESM2_ESM.pdf]

Description of Additional Supplementary Files

File Name: Supplementary Data 1
Description: Comparison of global confirmed cases of seasonal influenza FluNet data in the 16-month period from December 2018 to March 2020 (before the COVID-19 pandemic) against the 16-month period from April 2020 to July 2021 (during the COVID-19 pandemic).

File Name: Supplementary Data 2
Description: Circulating clades of seasonal influenza viruses observed from sequences in GISAID from April 2020 to July 2021.

File Name: Supplementary Data 3
Description: Transmission lineages of seasonal influenza viruses detected during 2020/2021 inferred using large-scale phylogenetic analysis of all HA sequences in GISAID from January 2018 to July 2021.

File Name: Supplementary Data 4
Description: Acknowledgements for influenza HA sequences downloaded from GISAID.
